# Opening Address

**Published:** 1846-09

**Authors:** 


*Opening Address.*—We expected to have been furnished with a copy of the opening address, delivered at the last meeting of the American Society of Dental Surgeons, for publication in the present number of the Journal, but as we have not yet received it, we hope Dr. Bridges will send it in time for the next.—*Balt. Ed.*

